# Review of patterns in homicides by sharp force: one institution’s experience

**DOI:** 10.1007/s12024-023-00576-8

**Published:** 2023-02-10

**Authors:** Petr Handlos, Tereza Švecová, Adéla Vrtková, Klára Handlosová, Marek Dokoupil, Ondřej Klabal, Juraj Timkovič, Matěj Uvíra

**Affiliations:** 1 Institute of Forensic Medicine, University Hospital Ostrava, Ostrava, Czech Republic; 2 Faculty of Electrical Engineering and Computer Science, Department of Applied Mathematics, VSB - Technical University of Ostrava, Ostrava, Czech Republic; 3 Department of the Deputy Director for Science, Research, and Education, University Hospital Ostrava, Ostrava, Czech Republic; 4 Faculty of Arts, Department of English and American Studies, Palacký University Olomouc, Olomouc, Czech Republic; 5 Clinic of Ophthalmology, University Hospital Ostrava, Ostrava, Czech Republic; 6 Faculty of Medicine in Hradec Kralove, The Fingerland Department of Pathology, Charles University, Hradec Kralove, Czech Republic

**Keywords:** Homicide, Blunt force trauma, Sharp force trauma, Defensive wounds, Assault escalation

## Abstract

This paper presents a retrospective review of patterns found in cases of homicides by sharp force over a 13-year period at the Department of Forensic Pathology of the Ostrava University Hospital, Czech Republic. The review summarizes all frequently discussed aspects of such cases including the number and localization of injuries, the presence of defensive wounds, the type of the offending weapon, the cause of death, the place of death, victims’ and perpetrators’ profiles, their relationship, or toxicological findings. Furthermore, special attention was paid to the evaluation of any accompanying blunt force trauma that may be indicative of an escalation of the assault. The set of data was statistically analyzed. Even though most of the results of this review are consistent with available published studies, noteworthy differences have emerged in some aspects such as the sex and age of the victims, the relationship between the number of injuries suffered and the victims’ sex, or the severity of alcohol intoxication in victims.

## Introduction

Sharp force injury fatalities are frequently encountered in forensic medicine. The manner of death in such cases is most frequently classified as homicide, followed by suicide and accident [1, 2]. Interestingly, a correlation can be seen between the gun control legislation existing in a specific country and the frequency of homicides involving sharp force. The stricter the gun laws and thus lower the availability of firearms, the higher the frequency of homicides by sharp force since sharp tools are the most easily available weapons. Taking the Czech Republic as an example of a country with rather strict gun control laws, the authors report that in their jurisdiction, sharp force fatalities represented 38.1% of all homicides between 2008 and 2020, making them the most frequent category of homicides, with firearm homicides merely accounting for 13.4%. This is in sharp contrast with the USA, where gun control laws are much more lenient, and firearm homicides accounted for 75% of all homicides [3].

This is in line with the data reported for other European cities and regions with strict gun control laws. The incidence of homicides involving sharp force as reported for some jurisdictions is as follows: 27% in Oslo (Norway), 31% in Lisbon (Portugal), 32.7% in Brescia County (Italy), 33% in Copenhagen (Denmark), and 37% in Stockholm area (Sweden) [4–8]. Given this context, this study is driven by the high frequency of such cases and their relevance for the community. The study discusses not only specific patterns of sharp force injuries in homicides, but also less frequently studied parameters such as the nature of the assault or perpetrators’ profiles. Special attention was paid to the evaluation of any accompanying blunt force injuries indicating the escalation of the assault prior to suffering the fatal sharp force injury or injuries.

## Methods

The autopsy files of the Department of Forensic Pathology of the Ostrava University Hospital, which provides autopsy services for the Moravian–Silesian Region with a population of approximately 1.19 million people, were searched for sharp force injury fatalities over a 13-year period from January 2008 to December 2020. Sharp force-related case files and autopsy reports were reviewed. The sex and age of the victims and perpetrators, the place of death, the nature of the assault, the type of sharp weapon used, the presence of clothes defects, the number and location of wounds, the presence and localization of defensive wounds, the cause of death, the toxicological findings of victims and perpetrators, and their relationship were summarized, and the corresponding statistical analysis was performed. For categorical variables, both absolute and relative frequencies are provided; for numerical variables, median values and ranges are provided. To analyze the data, the following statistical tests were used: the Mann–Whitney test, the Chi-Square, or the Fisher’s exact test. All statistical analyses were performed using the R software (version 4.0.2, www.r-project.org), and the significance level was set to 0.05.

## Results

Between 2008 and 2020, a total of 14,327 autopsies were performed at the Department of Forensic Pathology in Ostrava. Sharp force injury fatalities accounted for 167 cases including 91 cases of suicides, 71 cases of homicides, and 5 accidental cases.

Of the 71 cases, 43 victims were male and 28 female; 5 of all the victims were younger than 18 years. The age of the victims ranged from 0 to 79 years, and the median age of male and female victims was 47 years and 43 years, respectively.

The available evidence and police investigation indicate that 69 perpetrators were responsible for the total of 71 homicides. The lack of one-to-one correspondence is accounted for by the fact that in 4 cases, a single perpetrator killed two persons, and two acts were committed by 2 perpetrators. Of all the perpetrators, as many as 68 were caught, and only one remained unidentified. Of the identified perpetrators, a total of 72% were male and 28% were female; interestingly, only male perpetrators were responsible for the cases of double homicide. The median age of male and female perpetrators was 46 and 48 years, respectively. In 48% of the cases, the victim and the perpetrator were family members or partners; in 24%, they were friends; and in 28%, the perpetrator did not know the victim. Of the total of 71 homicides, 75% took place at home (houses, huts, etc.) and the rest, i.e., 25% of the homicides, took place at public areas including streets, pubs, or a swimming pool.

Of the total of 71 victims, 73% die immediately after assault, while 27% received CPR. Of those who received CPR, 37% were transported to the emergency department, 26% underwent abdominal and thoracic surgery, and 16% underwent thoracentesis.

In the majority of the cases (93%), the appearance of the wounds indicated the use of one or more knives, while only 7% of cases involved other sharp tools (a screwdriver, scissors, an axe). In 58% of the cases, the offending tool was found at the crime scene or in its proximity, and in mere 8% of cases, the offending tool was found to be stabbed in the victim’s body. In 82% of the cases, the victims were transported to the Department of Forensic Pathology in their clothing, while in 14% of the cases, the clothes were secured by the police at the crime scene or in the ambulance, and in only 4% of the cases, the clothes were not deliverable at all (in two cases, the clothes were destroyed by fire and one victim was murdered while taking shower; see Table 1).

**Table 1:** Summary of case details; the numbers represent the absolute frequencies and relative frequencies in percentages in the brackets

	*n* (%)
Sex of the victim	
Male	43 (61)
Female	28 (39)
Place of the homicide	
Home	53 (75)
Public areas	18 (25)
Offending tool	
Knife	66 (93)
Other	5 (7)
Offending tool found at the crime scene	
Yes	41 (58)
No	30 (42)
Clothing damage	
Yes	48 (68)
No	10 (14)
N/A	13 (18)

**Fig. 1:**
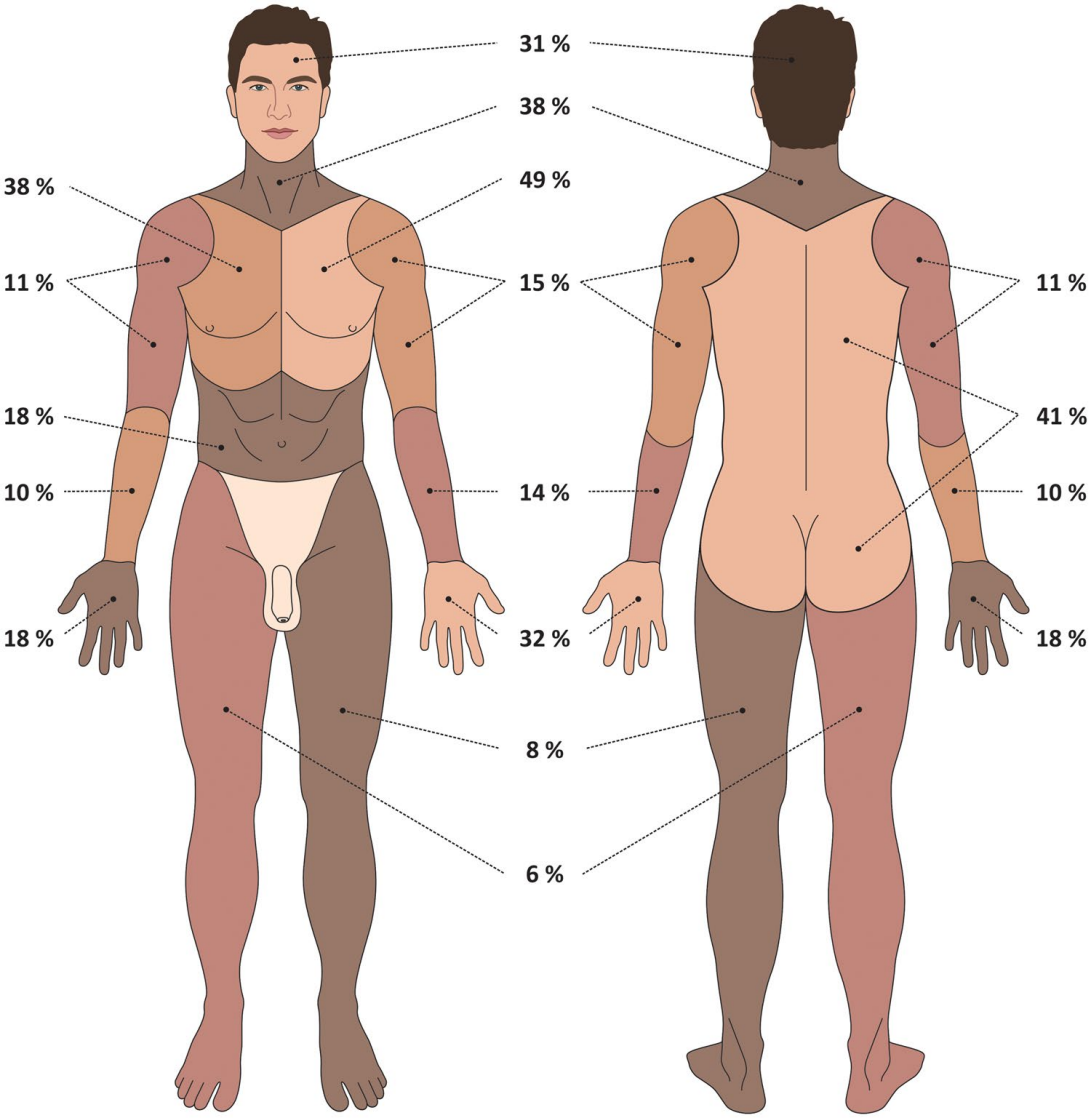
The location of wounds

Most of the homicide cases involved multiple sharp force injuries (79%), while a single sharp injury was found in 21% of the cases. The most frequently injured body area was the left portion of the chest (49%). Figure 1 shows the percentages of cases in which the respective body areas were injured (Fig. 1).

The most common cause of death was hemorrhagic shock (33%), followed by heart, neck, and intrathoracic vessel injury as shown in Table 2. These results are in agreement with the published studies.

**Table 2:** Cause of death; the numbers represent the absolute frequencies and relative frequencies in percentages in the brackets

	*n* (%)		
	Total (*n* = 71)	Men (*n* = 43)	Women (*n* = 28)
Cause of death			
Hemorrhagic shock	24 (33)	14 (32)	10 (34)
Fatal heart injury	19 (27)	11 (26)	8 (29)
Neck vessels injury	14 (20)	6 (14)	8 (29)
Thoracic vascular injury	9 (13)	8 (19)	1 (4)
Abdominal vascular injury	2 (3)	2 (5)	0 (0)
Vascular extremity injury	2 (3)	1 (2)	1 (4)
Cerebral contusions	1 (1)	1 (2)	0 (0)

This study aimed to determine the following:

1. The number of cases where the fatal sharp force assault was preceded by a blunt force assault, and the presence of blunt force defensive wounds in such cases.
2. The number of sharp force injuries sustained by the victims, including any defensive wounds, and the sex distribution thereof.
3. Positive toxicological findings in both perpetrators and victims, and the sex distribution thereof.

### Blunt force assault

This section analyzes the presence of injuries inflicted by kicks, punches, or a blunt tool as well as the presence of blunt force defensive wounds.

#### Presence of blunt force

Injuries located in the upper part of the skull or in central part of the face (i.e., orbits, nose, and lips) were attributed to blunt force assault. The injuries were caused by kicks, punches, or blunt tools such as a knife handle, barbell, hammer, and an axe butt. The data showed that blunt force assault preceded the sharp force assault in 27% of the cases as shown in Table 3. No significant difference in sex distribution was found.

**Table 3:** Presence of blunt force assault and defensive wounds; the numbers represent the absolute frequencies and relative frequencies in percentages in the brackets

	*n* (%)			
	Total (*n* = 71)	Men (*n* = 43)	Women (*n* = 28)	p*
Blunt force assault wounds				0.896
Yes	19 (27)	11 (26)	8 (29)	
No	49 (69)	31 (72)	18 (64)	
N/A	3 (4)	1 (2)	2 (7)	
Blunt force defensive wounds				0.716
Yes	16 (23)	11 (26)	5 (18)	
No	52 (73)	31 (72)	21 (75)	
N/A	3 (4)	1 (2)	2 (7)	
Presence of both blunt force assault and defensive wounds				0.425
Presence of both	12 (17)	9 (21)	3 (11)	
Only blunt force assault wounds	7 (10)	2 (5)	5 (18)	
Only blunt force defensive wounds	4 (6)	2 (5)	2 (7)	
Neither of the above	45 (63)	29 (67)	16 (57)	
N/A	3 (4)	1 (2)	2 (7)	

*The *p*-value of the Chi-square test of independence for contingency tables

#### Presence of blunt force defensive wounds

Injuries located in the dorsal part of the hand and ulnar side of the forearm were assessed as blunt force defensive wounds. The presence of such wounds was found in 23% of the cases (Table 3). No significant difference in sex distribution was found.

#### Presence of both blunt force and blunt force defensive wounds

The presence of both blunt force and blunt force defensive wounds was found in 21% of the male victims and 11% of the female victims (Table 3).

#### Blunt force assault and the sex of the perpetrator

Of note is the fact that the blunt force assault preceding the sharp force assault was only present in cases involving male perpetrators.

### Sharp force assault

This section evaluates the number of sharp force injuries and the sex distribution, as well as the presence of sharp force defensive wounds.

#### Type of wounds

The majority of the cases involved incised and stab wounds, while slash wounds were identified only in 2 cases.

#### Number of wounds

The number of sharp force injuries ranged from 1 to 68. The victims were assigned to three groups according to the number of injuries as shown in Table 4. The data reveal a significant difference in the number of injuries in male and female victims, with female victims suffering a higher number of injuries.

**Table 4:** Number of wounds and defensive wounds; the numbers represent the absolute frequencies and relative frequencies in percentages in the brackets if not specified differently

	Total (*n* = 71)	Men (*n* = 43)	Women (*n* = 28)	*p*
Number of wounds				0.010*
Median (Min–Max)	5 (1–68)	3 (1–49)	11 (1–68)	
1	15 (21)	13 (30)	2 (7)	
2–9	29 (41)	18 (42)	11 (39)	
10+	27 (38)	12 (28)	15 (54)	
Defensive wounds				0.322**
Yes	38 (54)	21 (49)	17 (61)	
No	30 (42)	21 (49)	9 (32)	
N/A	3 (4)	1 (2)	2 (7)	

*The *p*-value of the Mann–Whitney test; **The *p*-value of the Chi-square test of independence for contingency tables

#### Sharp force defensive wounds

**Fig. 2:**
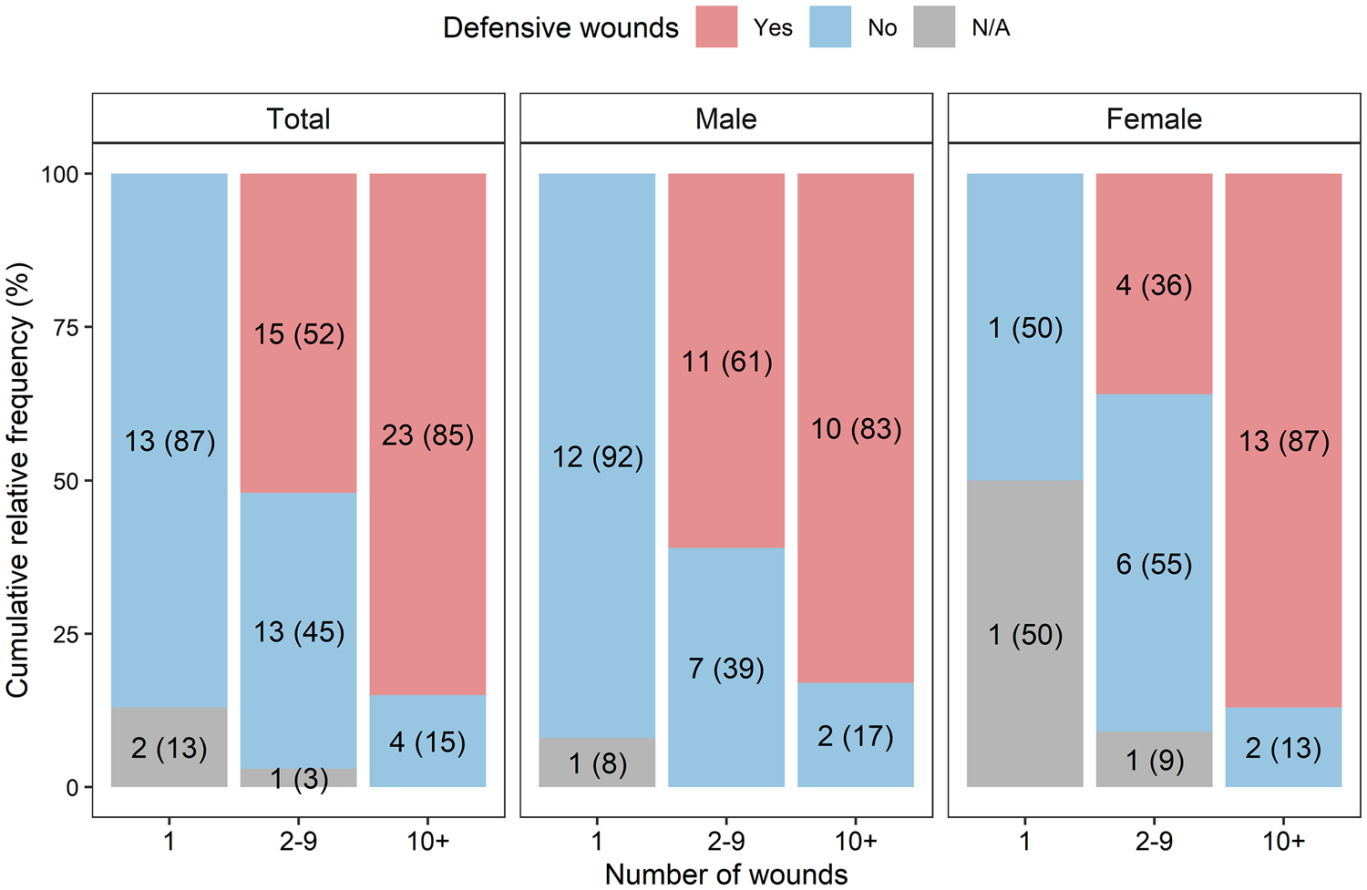
The relation between the number of wounds and defensive wounds in total (Fisher’s exact test, *p* = 0.002) and in male and female victims. The numbers represent the absolute frequencies and relative frequencies in percentages in the brackets

Sharp force defensive wounds were present in 54% of the cases; unfortunately, they could not be assessed in 4% of the cases owing to extensive thermal damage; no such wounds were present in the rest of the cases (Table 4). Figure 2 shows the number of sharp force defensive wounds indicating a significant relationship between the number of injuries and defensive wounds. The defensive wounds were most often localized on the left hands and forearms (Fig. 2). Limb penetration and partial finger amputation was present in only 8% of the cases.

### Toxicological findings in victims and perpetrators

All victims and most of the perpetrators were tested for the presence of alcohol and other relevant substances.

#### Toxicological findings in victims

Blood alcohol testing was performed in 99% of the victims. Alcohol intoxication was found in 59% of the victims, and the degree of intoxication is shown in Table 5. A statistically significant difference was found between male and female victims, with male victims being intoxicated more frequently (Table 5). Furthermore, cannabis intoxication was found in 1 case, and combined cannabis and methamphetamine intoxication was found in 1 case.

**Table 5:** Level of alcohol intoxication in victims; the numbers represent the absolute frequencies and relative frequencies in percentages in the brackets

	*n* (%)			
	Total (*n* = 71)	Men (*n* = 43)	Women (*n* = 28)	p*
Level of alcohol intoxication in victims				0.003
Sober	28 (40)	9 (21)	19 (67)	
Mild intoxication	5 (7)	4 (9)	1 (4)	
Increased intoxication	8 (11)	5 (12)	3 (11)	
Severe intoxication	14 (20)	11 (26)	3 (11)	
Life threatening intoxication	15 (21)	13 (30)	2 (7)	
Unknown	1 (1)	1 (2)	---	

*The p-value of the Chi-square test of independence for contingency tables

#### Toxicological findings in perpetrators

If possible, the perpetrators were also tested for the presence of alcohol. Unfortunately, the testing could not be performed in 24% of the cases. In cases where the perpetrator was arrested within short time after committing the crime, a retrospective calculation was performed to determine the blood alcohol level at the time of committing the crime (the applied rate of decline after the reaching the peak of the curve was 0.012–0.020%/h). The level of alcohol intoxication is shown in Table 6. No statistically significant difference was found between male and female perpetrators (Table 6). In addition, three perpetrators were tested positive for cannabis, one for methamphetamine and one was both cannabis and toluene.

**Table 6:** Level of alcohol intoxication in perpetrators; the numbers represent the absolute frequencies and relative frequencies in percentages in the brackets

	*n* (%)			
	Total (*n* = 68)	Men (*n* = 49)	Women (*n* = 19)	p*
Level of alcohol intoxication in perpetrators				0.816
Sober	22 (33)	15 (31)	7 (36)	
Mild intoxication	1 (1)	1 (2)	---	
Increased intoxication	7 (10)	4 (8)	3 (16)	
Severe intoxication	19 (28)	13 (27)	6 (32)	
Life threatening intoxication	3 (4)	3 (5)	---	
Unknown	16 (24)	13 (27)	3 (16)	

*The *p*-value of the Fisher’s exact test

## Discussion

In general, the results of this study, such as the number of male victims being higher than female victims, are consistent with available studies dealing with sharp force injury fatalities [1, 5, 9]. Since this study aims to be comprehensive and include as many factors as possible, it proved to be rather challenging to find similar studies for other jurisdictions with data for all criteria reported. Therefore, this section will discuss the results and compare them with other published studies, as far as data availability permits.

Unlike in other studies where the percentage of female victims ranged from 30 to 35% [5, 10–12], it was slightly higher in this study, namely 39%. In general, the male to female ratio in the published studies varies from 2 to 5, with the exception of the study by Belghith et al. involving Aboriginal population in Central Australia where female victims accounted for 53% of all the victims [13–15]. Such high number of female victims in Aborigines population could be explained by the use of traditional punishment, which is still practiced in Central Australia [15].

While the age of the victims in developing countries usually ranges from 20 to 30 years, data from developed countries show a significantly higher average age of the victims [1, 11–13, 16, 17]. This difference could be accounted for by different socioeconomic situations, differences in lifestyle, and different life expectancy in these countries.

As reported in the results, 73% of the victims die immediately after assault owing to the extent and lethal nature of the sustained injuries. Only 10% of all the victims were admitted to hospital. The victims who stayed alive long enough to be transported to hospital sustained penetrating stab wounds with no severe internal organ damage.

For illustrative purpose, the typical case scenario can be summarized as follows: A male perpetrator assaulting his victim at home using a knife. The victim and the perpetrator were related or knew each other. This scenario is also consistent with the results of other studies from Europe and Japan [5, 12, 18]. Of note is the fact that in Tunisia, sharp force homicides were most commonly committed in public places (62.4%) which is in contrast with both the majority of the published studies and with the study presented in this paper [13].

Published studies report that the offending weapon was found at the crime scene or in the proximity of the crime scene in 49–53%. In our cohort, the frequency of the presence of the weapon at the crime scene was slightly higher (58%), and in 8% of the cases, the weapon was found to be stabbed in the victim’s body. This finding slightly differs from the studies by Terranova or by Thomsen et al. who report the offending weapon stabbed in the victim’s body in 3.3% and 4.5%, respectively [12, 19]. The frequency of clothes defects in cases of sharp force homicides as reported ranges from 71 to 89% [6, 19, 20]. In this study, clothes defects were seen in 83% of the cases; the more wounds suffered, the more frequent the clothes damage. In cases of single wounds, clothes defects were found in 83% of the cases, which is consistent with the findings reported by Burke et al. who reported clothes defects in 85% of their cases of single stab wounds [21].

The majority of the cases involved multiple incised and/or stab wounds, while slash wounds were found only rarely, which corresponds to results of the published studies [5, 11, 22]. This is not surprising given that a household is the most common place of assault in developed countries, where an easy knife availability can be assumed. A single stab wound was found in 21% of the cases. The published data regarding single stab wounds vary widely; Vassalini et al. found a single stab wound only in 9.8% of their cases, while Thomsen et al. report the presence of a single stab in 18.9% of their cases [1, 12]. In the presented cohort, a single stab wound was found predominantly in male victims, while in female victims, a single stab wound was found rarely.

Previous studies have identified the thorax/neck to be the most common site of sharp force injuries suffered in homicides, with the heart and the large vessels being the most frequently injured organs, which is in agreement with the findings of the present study [12, 23–26]. Such findings could be well explained by the fact that the victim and the assailant were facing each other at the moment of the assault, and possibly the assailant’s general knowledge of human anatomy and the position of the vital organs and vessels.

The study also aimed to determine whether the sharp force assault was preceded by blunt force assault. Such use of the blunt force may be expected in cases of conflict escalation when the perpetrator believes to be stronger than the victim. Such assaults were committed only by male perpetrators against both male and female victims, with little difference across the sexes. To be precise, 26% of male victims and 29% of female victims suffered such injuries before the fatal sharp force assault, which is contrary to what one would expect, i.e., higher incidence of such injuries in female victims due to their physical condition. The frequency of blunt force defensive wounds did not show a significant difference across the sexes. This corresponds well with the findings by Rogde et al. who observed that the incidence of additional violence does not differ between the sexes [5].

With respect to sharp force assault, there was a statistically significant difference in the number of sharp force injuries in male and female victims, with male victims suffering fewer injuries, including defensive wounds. Such observation is consistent with other published studies [1, 8, 12, 20].

The sharp force defensive wounds were found in 54% of all cases. Previous studies have shown the presence of such defensive wounds in 31–64% of the cases of homicidal deaths from sharp weapon injury [12, 19, 20, 27–31]. The percentage increases if the number of stab wounds is higher. In our cohort, the presence of defensive wounds was found in 85% of the victims who suffered more than 10 stab wounds. Similarly, Thomsen et al. report the presence of defensive wounds in 76.2% of the cases where the victim suffered more than 10 stab wounds [12]. In the studied cases, the presence of defensive wounds was found in 49% of male victims and in 61% of female victims. The difference in frequency of the presence of defensive wounds between male and female victims may possibly be explained not only by the number of the suffered wounds but also by higher blood alcohol level in male victims, which could compromise both their self-defense as well as motor coordination.

The most frequent sites of defensive wounds included fingers, palms, and the dorsal side of the hands, with the dominance of left hand. This conclusion is in contrast with published studies which report the palmar site of the right hand to be the most frequently injured part of the body during defense [1, 6, 26]. There was a comparatively high incidence of injuries in left arm and shoulder, i.e., in sites adjacent to the left side of the thorax, which may be explained by the fact that such injuries may either qualify as defensive wounds or primary injuries caused by sharp force assault against the torso, which makes their interpretation challenging. The results showed that the higher the number of sharp force injuries, the higher the number of defensive wounds. Defensive wounds were present in all victims who suffered more than 10 sharp force injuries and died due to hemorrhagic shock (i.e., there was no injury directly causing the death). A special case was a victim who suffered 72 sharp force injuries predominantly in the area of the neck and upper part of the thorax, but no defensive wounds were present. This case involved a brutal assault, and it can be assumed that some of the initial injuries were fatal, and thus prevented the victim from defending herself.

Finally, the study also assessed the intoxication of both victims and perpetrators. In terms of alcohol intoxication, zero blood alcohol level was found in 67% of female victims and only in 21% of male victims. This is in agreement with results from Scandinavia published by Rogde et al. who found that 35% of the male and 63% of the female victims had zero blood alcohol level [5]. In most cases, the blood alcohol level was within the level of severe and life-threatening alcohol intoxication (BAC, i.e., > 0.31%). This is in contrast to the findings by Belghith et al. who report the frequency of alcohol-intoxicated victims in Tunisia of only 4.2% [13]. These difference may be explained by higher alcohol consumption by Europeans compared to other countries, especially Islamic countries [32]. Intoxication involving other substances was rather rare. The perpetrators were tested for their blood alcohol level only in 76% of the cases, and severe or life-threatening intoxication was found in approximately one-third of male and female perpetrators. Intoxication involving other substances was negligible.

## Conclusions

In many aspects, this study has confirmed what has been published on sharp force homicides earlier. What differs is the higher incidence of female victims as well as higher age of both male and female victims. Furthermore, the number of sharp force injuries suffered by female victims was significantly higher than that suffered by male victims. No statistically significant difference was found between the number of blunt force injuries suffered by male and female victims before the fatal sharp force assault. Such injuries were present in approximately 25% of the victims, and the cases usually involved a male perpetrator. Contrary to published studies, a higher incidence of severe or life-threatening alcohol intoxication in male victims was found. In conclusion, this retrospective study shows that while many aspects of sharp force homicides tend to be universal, there may be slight differences in results when different countries are contrasted and files of different departments and time periods are analyzed. When such differences exist (e.g., the age of the victims), they may sometimes be explained by the social and economic situation in the respective countries and regions.

## Key points

1. Homicides by sharp force are the most frequent type of homicides in the Czech Republic.
2. The presence of both blunt force and sharp force trauma may be indicative of assault escalation.
3. Alcohol intoxication is more frequent in male victims and male perpetrators.
4. The sex and age of the victims may be related to the socioeconomic situation of the country.

## Data Availability

Data for this study can be requested by contacting the corresponding author.
